# Mentoring is in the ‘I’ of the beholder: supporting mentors in reflecting on their actual and preferred way of mentoring

**DOI:** 10.1186/s12909-022-03690-3

**Published:** 2022-08-23

**Authors:** Lianne M. Loosveld, Erik W. Driessen, Eline Vanassche, Anthony R. Artino, Pascal W. M. Van Gerven

**Affiliations:** 1 School of Health Professions Education, Department of Educational Development & Research, Faculty of Health, Medicine and Life Sciences, Maastricht University, Universiteitssingel 60, 6229 ER Maastricht, The Netherlands; 2 Faculty of Psychology and Educational Sciences, University of Leuven, Campus Kulak, Etienne Sabbelaan 51, P.O. Box 7654, 8500 Kortrijk, Belgium; 3 Department of Health, Human Function, and Rehabilitation Sciences, School of Medicine and Health Sciences, The George Washington University, 2600 Virginia Avenue NW, Suite 104, Washington, DC, USA

**Keywords:** Mentoring, Critical reflection, Faculty development, Personal interpretative framework

## Abstract

**Background:**

An important strategy to support the professional development of mentors in health professions education is to encourage critical reflection on what they do, why they do it, and how they do it. Not only the ‘how’ of mentoring should be covered, but also the implicit knowledge and beliefs fundamental to the mentoring practice (a mentor’s personal interpretative framework). This study analyzed the extent to which mentors perceive a difference between how they actually mentor and how they prefer to mentor.

**Methods:**

The MERIT (MEntor Reflection InstrumenT) survey (distributed in 2020, *N* = 228), was used to ask mentors about the how, what, and why of their mentoring in two response modes: (1) regarding their actual mentoring practice and (2) regarding their preferred mentoring practice. With an analysis of covariance, it was explored whether potential discrepancies between these responses were influenced by experience, profession of the mentor, and curriculum-bound assessment requirements.

**Results:**

The averaged total MERIT score and averaged scores for the subscales ‘Supporting Personal Development’ and ‘Monitoring Performance’ were significantly higher for preferred than for actual mentoring. In addition, mentors’ experience interacted significantly with these scores, such that the difference between actual and preferred scores became smaller with more years of experience.

**Conclusions:**

Mentors can reflect on their actual and preferred approach to mentoring. This analysis and the potential discrepancy between actual and preferred mentoring can serve as input for individual professional development trajectories.

**Supplementary Information:**

The online version contains supplementary material available at 10.1186/s12909-022-03690-3.

## Introduction

Mentors in health professions education are faculty who support their mentees’ personal and professional development [1–6]. They can influence the career of the next generation of healthcare providers, making the professional development of mentors a key priority for health professions programs. An important strategy to support mentors’ professional development is encouraging critical reflection on what they do, why they do it, and how they do it [7–14]. Research on reflection in and beyond health professions has convincingly shown that the connection between mentors’ representations of their mentoring practice and their actual enactment of practice is rather loose [13, 14]. There often is a gap between what practitioners want or intend to do in practice and what they actually do [15]. Research suggests a myriad of explanations for these gaps, including institutional, curricular or collegial role expectations that conflict with mentors’ personal understandings of good mentoring [16, 17], but also routinized individual and group behaviors and a lack of understanding of the beliefs that tacitly underpin practice [18, 19]. Critical reflection is crucial for mentors to identify the beliefs governing their actions, critically examine them, and explore alternatives for practice. It might help mentors to map and better understand the gap between the expressed and the realized, and if desirable, also close this gap [20, 21]. It is, however, not self-evident that mentors, often supporting their mentees to become reflective practitioners, are proficient themselves at reflecting on their experiences [1, 7]. Both the readiness and the ability to critically reflect on one’s own mentoring practice and the beliefs and knowledge underpinning this practice differ between mentors [7, 10], indicating a need for supporting mentors in this reflection process as part of their professional development.

To help mentors make the “what, why, and how” of their actual practice explicit, and explore the implicit system of knowledge and beliefs underpinning practice, we developed a survey called MERIT: MEntor Reflection InstrumenT [4]. The intent of the MERIT is not to measure underlying psychological constructs, but rather to promote mentors’ reflection on their role. Its development resulted in the identification of four ‘focus points’ in mentors’ reflection on their mentoring practice: (1) supporting personal development, (2) modelling professional development, (3) fostering autonomy, and (4) monitoring performance [4].

The MERIT draws on research in the field of mentoring as well as our own empirical work on mentors’ *personal interpretative framework*. Kelchtermans [22] describes this framework as a lens that teachers use to interpret and interact with their professional context. At the same time, the lens is influenced by that professional context too. It includes two dimensions with multiple underlying components, allowing for a more dynamic understanding of mentors’ sense of self than the related notion of teacher identity. The first dimension of the personal interpretative framework is *professional self-understanding*. This is the understanding mentors have of themselves as mentors at a certain point in time (the ‘what’ and ‘why’ of their mentoring). The second dimension, *subjective educational theory*, encompasses a mentor’s personal system of knowledge and beliefs about the way they mentor (the ‘how’) [22]. This multidimensional, dynamic view closely aligns with how van Lankveld, Thampy, Cantillon, Horsburgh and Kluijtmans [23] conceptualize teacher identity: as “both an understanding and as a presentation of oneself, shaped and reshaped in constant dialogue between a person and their social environment” (p. 2). Along similar lines, the personal interpretative framework is dynamic, rather than static, as it results from the meaningful interactions between mentors and their professional working context.

In the current article, we report on additional data about ‘preferred mentoring’ gathered during the MERIT development study [4]. With this additional data from this same sample of mentors we investigated the extent to which they experienced a gap between their actual and preferred mentoring. We base our analysis on the following two research goals: First, we evaluated whether mentors experience a discrepancy between their actual and preferred approach to mentoring. Second, we explored whether any discrepancy between actual and preferred mentoring is associated with mentors’ experience measured in years, their profession (e.g., educationalist, researcher, or physician), or the requirement to assess the performance of their mentees (e.g., a mentee’s portfolio in a programmatic assessment setting) [3, 24–28].

## Materials and methods

### Respondents

**Fig. 1:**
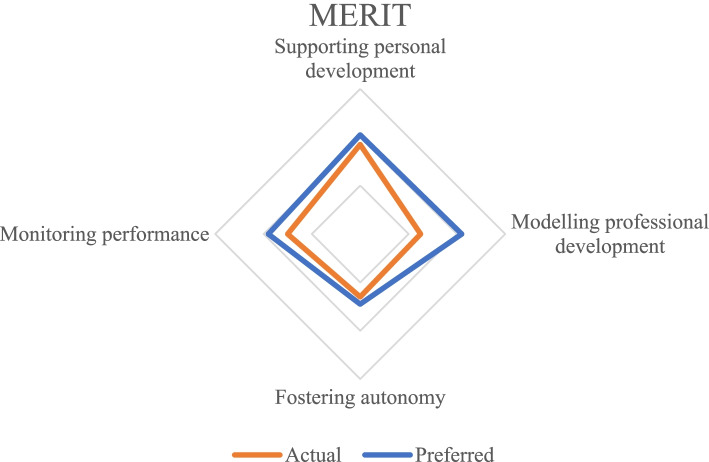
A simulated radar chart of the MERIT data. The depicted data do not belong to any of the respondents in this study and were generated for illustrative purposes only

We invited mentors in health professions education to participate. In this article, our target population of mentors in health professions education is defined as faculty members who have a formal mentoring relationship with one or multiple (under)graduate students. The focus of this relationship is on supporting competence development and stimulating reflection (after Nicholls [2]). Respondents mentoring postgraduate students or mentoring outside the domain of health professions education were excluded from the sample, but no further exclusion criteria applied. Respondents were provided with a participant information letter, and a signed online informed consent was obtained from all respondents. All mentors who indicated that they were interested in receiving their survey results were sent an e-mail with an explanation and a radar chart (Fig. 1), summarizing their individual results. The chart presented the difference between their actual and their preferred mentoring through colored lines.

### Survey information

An extensive description of the design, distribution, and analysis of the psychometric properties of the survey can be found in Loosveld, Van Gerven, Driessen, Vanassche, Artino [4]. The survey was designed based on previous qualitative work with mentors in health professions education [28] and an extensive review of the literature on mentoring. It has been pre-tested and piloted, and the internal structure and reliability of the final survey have been assessed based on responses from an international sample of mentors [4]. The MERIT is composed of 20 items that use a five-point, Likert-type response scale: ‘this item is’ (1) not at all true of me – (2) slightly true of me – (3) somewhat true of me – (4) mostly true of me – (5) completely true of me [29]. A higher score thus indicates that mentors identify more with that particular item.

### Sampling and survey distribution

A link to the online MERIT survey, which was hosted on Qualtrics (Provo, Utah), was distributed via Twitter accounts of the researchers (around 4000 cumulative followers), LinkedIn (around 800 connections), and via 128 e-mails to contact persons between November 2019 and March 2020. Because of this distribution via contact persons and social media, the exact overall denominator was unknown, as a result of which it was impossible to calculate the overall response rate. As this study did not intend to characterize a population, the lack of response rate was considered less problematic [30].

### Procedure

Upon signing informed consent, mentors entered an online survey environment where they were presented with each MERIT item twice. In each of those two instances, the question had to be answered in a specific response mode: the first time the respondents reported about their own *actual* mentoring practice and the second time, immediately after the first time, respondents were asked to envision their *preferred* mentoring. We included the following instruction to explain the two response modes (Table 1):

**Table 1:** Written instruction explaining *actual* and *preferred *response mode

Considering how you mentor, how true or untrue are these following 20 statements for you? In the first set of answers, think about how you actually, currently act as a mentor, not how you ideally would want to or should act (that is, not based on either theory or how your colleagues mentor others). The second set of answers allows you to indicate how you would prefer to mentor. The answers to these two questions can be the same, but there can also be a difference between them. There are, however, no wrong answers to any of these questions.	

Eight demographic questions and two open-ended questions about the content and design of the survey concluded the survey. The factor structure within the set of survey items was previously determined via Principal Access Factoring and the internal consistency reliability of the subscale scores evaluated using Cronbach’s alpha [4]. Based on the Principal Access Factoring, the four subscales of the MERIT were determined to be: (1) *supporting personal development*, with survey items on the personal development of mentees, (2) *modelling professional development*, with items about providing insight on how academia works (3) *fostering autonomy* about advice-seeking and problem-solving, and (4) *monitoring performance*, about accessing and understanding performance data. An extensive description of the design, distribution, and analysis of the psychometric properties of the survey can be found in Loosveld, Van Gerven, Driessen, Vanassche, Artino [4] .

### Analysis

To reach our current research objectives, we ran one-way analyses of covariance (ANCOVA) with Response Mode (levels: Actual, Preferred) as the within-groups independent variable. The dependent variables were the average score on the entire MERIT survey, as well as average scores for the four subscales, based on MERIT factors. We included three covariates in our model: (1) Experience, (2) Main Profession, and (3) Assessment. Experience was the amount of mentoring experience in years. Main Profession was defined as the profession that mentors primarily identified with (Basic scientist, Researcher, Physician, Teacher/Educator, Educationalist, Sociologist, Psychologist, PhD-candidate, Other). Assessment, finally, indicated whether mentors were required to assess their mentee or not (Yes, No, Do not know). SPSS statistical software, version 25 (IBM Corporation, New York) and Microsoft Excel 2016 (Microsoft Corporation, Redmond, Washington) were used for data analysis and data management.

### Ethical approval

This research was approved by the Maastricht University Ethics Review Committee (UM-REC), file number: FHML-REC/2019/033, October 1, 2019.

## Results

After removing the data of four respondents mentoring outside the field of health professions education, 228 fully completed surveys remained for analysis.

### Demographics

Our sample consisted of 77 (34%) mentors who identified as men and 148 (65%) who identified as women. One mentor indicated ‘other’ as their gender and two other mentors did not identify their gender (1%). The mean age of the respondents was 46.4 years (range = 26–72 years; three mentors did not reveal their age). As can be seen from Table 2, some mentors in our sample indicated being quite experienced, but given that it is not uncommon for health professionals to continue mentoring well after their retirement [31, 32], we did not consider their responses as inaccurate or erroneous. Since we did not require a specific minimum or maximum number of years of mentoring experience in order to participate in our study, we had no way to control how many junior or senior mentors participated in our study. Given that we invited mentors from the health professions education domain, it is not surprising that there is a relatively large proportion of mentors (35.5%) who identified ‘physician’ as their main profession. Additional information on mentors’ professional working context and personal demographics can be found in Table 2.

**Table 2:** Features of professional working context and personal demographics of the 228 MERIT survey respondents

Variable	No. of respondents (% of 228)
**Initial training of mentor**	
Medicine	121 (53.1%)
Educational Sciences	41 (18.0%)
Health Sciences	35 (15.4%)
Psychology	24 (10.5%)
Biomedical Sciences	18 (7.9%)
Basic Sciences	13 (5.7%)
Social Sciences	10 (4.4%)
Allied Health Professions	8 (3.5%)
Public Health	6 (2.6%)
Nursing Sciences	2 (0.9%)
Pharmacy	2 (0.9%)
Other	22 (9.6%)
**Current main profession**	
Physician	81 (35.5%)
Researcher	45 (19.7%)
Teacher/Educator	42 (18.4%)
Educationalist	23 (10.1%)
PhD Candidate	16 (7.0%)
Basic Scientist	5(2.2%)
Other	16 (7.0%)
**Educational Program in which mentor mentors**	
Medicine	137 (60.1%)
Health Sciences	33 (14.5%)
Educational Sciences	22 (9.6%)
Biomedical Sciences	19 (8.3%)
Allied Health Professions	5 (2.2%)
Pharmacy	2 (0.9%)
Public Health	1 (0.4%)
Dentistry	1 (0.4%)
Other	8 (3.5%)
**Country in which mentor mentors (per continent)**	
Europe	168 (73.3%)
North America	43 (18.9%)
Australia	8 (3.5%)
Asia	6 (2.6%)
Africa	3 (1.3%)
**Years of mentoring experience** ^**a**^	
0–5	99 (43.4%)
6–10	64 (28.1%)
11–15	31 (13.6%)
16–20	14 (6.1%)
21–25	13 (5.7%)
26–30	7 (3.1%)
31–35	2 (0.9%)
36–40	1 (0.4%)
41–45	0 (0.0%)
46–50	0 (0.0%)
51–55	0 (0.0%)
56–60	1 (0.4%)
**Mentor assesses mentee**	
Yes	180 (78.9%)
No	41 (18.0%)
Don’t know	7 (3.1%)

^a^ For the sake of brevity, this variable is shown in categorical units. It is analyzed as a continuous variable

### Total MERIT score

The results of the ANCOVA yielded a significant main effect of Response Mode, *F*(1, 224) = 15.20, *p* < .001, η_p_^2^ = .064, indicating that the average total MERIT score was higher in the Preferred (*M* = 4.12, *SD* = .34) than in the Actual (*M* = 3.96, *SD* = .36) response mode (see Tables 3 and 4). The covariate Experience did not have a significant main effect on the total MERIT score, *F*(1, 224) = 1.38, *p* = .241, η_p_^2^ = .006, and neither did the other two covariates, Main Profession and Assessment (*F*s < 1).

**Table 3:** Mean MERIT scores on total and subscale level

	*M*	*SD*
Total MERIT score: Actual	3.96	0.36
Total MERIT score: Preferred	4.12	0.34
Supporting Personal Development: Actual	4.29	0.55
Supporting Personal Development: Preferred	4.53	0.45
Modelling Professional Development: Actual	3.68	0.58
Modelling Professional Development: Preferred	3.67	0.64
Fostering Autonomy: Actual	3.70	0.71
Fostering Autonomy: Preferred	3.76	0.74
Monitoring Performance: Actual	4.02	0.59
Monitoring Performance: Preferred	4.36	0.55

**Table 4:** Main effects of response mode and covariates on total MERIT score

	*F*	*p*	η_p_^2^
Response Mode	15.20	.000^***^	.064
Experience	1.38	.241	.006
Main profession	0.00	.960	.000
Assessment	0.84	.359	.004

*** *p* < .001

**Fig. 2:**
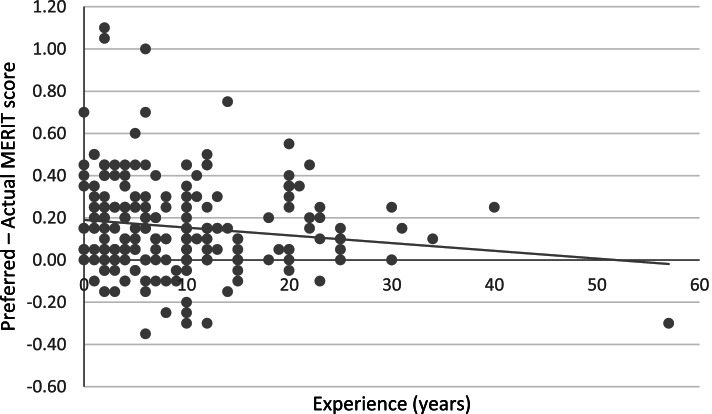
Difference in the overall mean score on the MERIT between the Actual and Preferred Response Mode (i.e., Preferred – Actual) as a function of Experience in years

There was however, a significant Response Mode × Experience interaction, *F*(1, 224) = 4.76, *p* = .030, η_p_^2^ = .021, suggesting that the effect of Response Mode – that is, the discrepancy between Actual and Preferred MERIT scores – became smaller with more years of experience (see Fig. 2 for a representation of the interaction pattern). The other three covariates did not show significant results (see Table 5).

**Table 5:** Interactions between Response Mode and the three covariates for total MERIT scores

Dependent variable	Interaction	*F*	*p*	η_p_^2^
Total MERIT score	Response Mode × Experience	4.76	.030^*^	.021
	Response Mode × Main Profession	0.50	.481	.002
	Response Mode × Assessment	0.39	.536	.002

* *p* < .05

### MERIT subscale scores

The ANCOVAs of two of the four factors, Supporting Personal Development and Monitoring Performance, yielded significant main effects of Response Mode. Results for Supporting Personal Development were *F*(1, 224) = 13.75, *p* < .001, η_p_^2^ = .058, indicating that mentors’ score on this factor was higher for Preferred than for Actual mentoring. For Monitoring Performance, mentors’ Preferred scores were again higher than Actual scores, *F*(1, 224) = 13.01, *p* < .001, η_p_^2^ = .055 (see Table 6). The covariate Main Profession was found to have a significant main effect on Fostering Autonomy, *F*(1, 224) = 12.99, *p* < .001, η_p_^2^ = .055. The other covariates did not show main effects on any of the four factors (*F*s < 1) (see Table 6 for a complete overview of main effects of the covariates).

**Table 6:** Main effect of Response Mode and covariates on MERIT subscale scores

Dependent variable	Main effect	*F*	*p*	η_p_^2^
Supporting Personal Development	Response Mode	13.75	.000^***^	.058
	Experience	0.60	.438	.003
	Main profession	0.02	.886	.000
	Assessment	0.00	.962	.000
Modelling Professional Development	Response Mode	0.01	.918	.000
	Experience	0.04	.840	.000
	Main profession	0.76	.384	.003
	Assessment	0.88	.349	.004
Fostering Autonomy	Response Mode	1.79	.182	.008
	Experience	1.67	.197	.007
	Main profession	12.99	.000^***^	.055
	Assessment	0.05	.821	.000
Monitoring Performance	Response Mode	13.04	.000^***^	.055
	Experience	2.91	.089	.013
	Main profession	2.09	.149	.009
	Assessment	3.98	.047	.017

*** *p* < .001

**Fig. 3:**
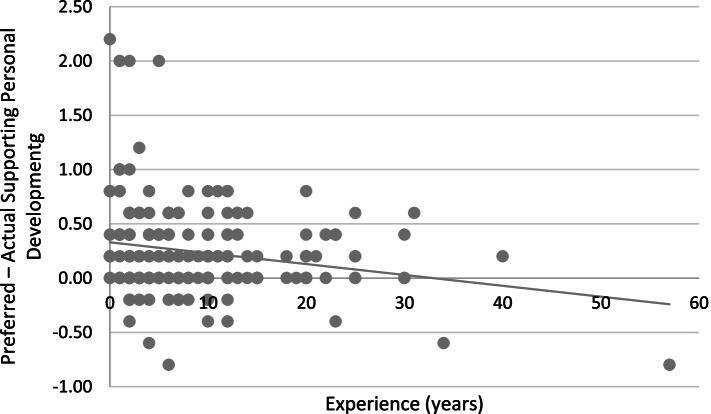
Difference in the mean score on the MERIT between the Actual and Preferred Response Mode (i.e., Preferred – Actual) on the subscale Supporting Personal Development as a function of Experience in years

Response Mode did not interact with Main Profession or Assessment (*F*s < 1), suggesting that these covariates did not affect the discrepancy between Actual and Preferred MERIT scores (see Table 7). For Supporting Personal Development there was a significant Response mode × Experience interaction, *F*(1, 224) = 10.55, *p* = .001, η_p_^2^ = .045, again suggesting that the effect of Response Mode, on the level of Supporting Personal Development, became smaller with more years of Experience (see Fig. 3 for a representation of the interaction pattern).

**Table 7:** Interactions between Response Mode and the three covariates on MERIT subscale level

Dependent variable	Interaction	*F*	*p*	η_p_^2^
Supporting Personal Development	Response Mode × Experience	10.55	.001^**a^	.045
	Response Mode × Main Profession	0.86	.354	.004
	Response Mode × Assessment	0.64	.423	.003
Modelling Professional Development	Response Mode × Experience	0.18	.673	.001
	Response Mode × Main Profession	0.09	.767	.000
	Response Mode × Assessment	0.00	.992	.000
Fostering Autonomy	Response Mode × Experience	0.84	.362	.004
	Response Mode × Main Profession	0.10	.754	.000
	Response Mode × Assessment	2.95	.087	.013
Monitoring Performance	Response Mode × Experience	4.33	.039^*^	.019
	Response Mode × Main Profession	0.56	.455	.002
	Response Mode × Assessment	3.22	.074	.014

* *p* < .05, ** *p* < .01. ^a^ Remains significant after Bonferroni correction

A significant Response mode × Experience interaction in that same direction was found for the subscale Monitoring Performance, *F*(1, 224) = 4.33 *p* = .039, η_p_^2^ = .019, although this interaction did not survive Bonferroni correction. No further interactions between response mode and covariates were found. Table 7 includes the full overview of interactions.

## Discussion

Findings from this study suggest that the mentors in our sample perceive a discrepancy between their actual and preferred mentoring. Moreover, mentoring experience significantly moderated this discrepancy: The more years of experience as a mentor, the smaller the discrepancy became. This interaction effect appeared to be driven by responses on the subscale Supporting Personal Development.

It is important to note that we did neither intend to make evaluative statements about mentoring capabilities, nor did we try to uncover the reason behind discrepancies between one’s actual and preferred mentoring. Moreover, identified discrepancies between actual and preferred mentoring do not imply that someone is not a good mentor. Rather these discrepancies may indicate conflicting narratives – for example, between professional self-understanding and curriculum requirements –, which could hamper mentors to put their personal knowledge and beliefs into practice [33–35]. Prior research has shown the potentially detrimental effects of not being able to act according to one’s personal beliefs for mentors’ job motivation and collegial position [13, 16, 36].

Based on these findings, we believe that the merit of the MERIT survey for mentors lies in offering support during their professional development. Critical reflections on experiences from their daily practice can help mentors to identify and prioritize learning needs [15, 37, 38], thereby serving as an entry point for their professional development [12]. This enables mentors to acquire, refine, or broaden their mentor-specific knowledge and skills [28, 39–43]. We therefore argue that not only students [44], but also faculty in medical education should be supported in the reflective process that is foundational to their professional development. Without critical reflection on the how, what, and why of mentoring, faculty development may be nothing more than transferring custom practices and tricks of the trade, without thinking through why, for whom, and under what conditions these approaches (do not) work [2, 45].

A limitation to this study is that we had little means to control who filled out the survey. Even though we asked mentors to respond only when they met our inclusion criteria and we examined the responses for mentors who did not meet the inclusion criteria, we cannot be sure that all respondents indeed fit our description of mentors in health professions education. In addition, despite our efforts to distribute the survey globally, the majority of our respondents fulfilled their mentoring role in Europe (73.3%) or North America (18.9%). Therefore, our sample may not reflect a worldwide representation of mentors in health professions education and we cannot rule out the influence of, for example, local administrative rules and regulations. However, given the context specificity of the personal interpretative framework, we argue that an accurate representation of how individual mentors perceive their mentoring only exists within the specific professional working context of that mentor.

Another limitation of this study is that we were not able to analyze how mentors interpreted or explained their reflections. Follow-up research could therefore take a more explanatory approach, where mentors are asked to reflect on their mentoring practice and then, together with an interviewer, explore their answers and discuss how those answers shape their personal interpretative framework. Because of our quantitative approach, we also do not know whether there are other factors that might influence mentors’ actual and preferred mentoring. Given the personalized and contextualized nature of mentoring, this is an avenue that warrants further exploration.

## Conclusion

The perceived discrepancy between actual and preferred mentoring of the mentors in our sample is influenced by their years of experience: More experienced mentors perceive a smaller discrepancy between their actual and preferred mentoring. This discrepancy could guide faculty development initiatives that involve active and collaborative formats to help mentors discuss, reinforce, and challenge their personal interpretative framework.

## Supplementary Information

**Additional file 1.**

## Data Availability

The survey used for this study is available as Supplemental Online Material (File 1). The dataset generated and analyzed in the current article is not publicly available to guard anonymity of the respondents, but it is available from the corresponding author on reasonable request.
